# Wild *Daucus carota* Subsp. *maximus*: Phytochemicals, Bioactivities, and Pharmacokinetic Insights for Future Food and Therapeutic Applications

**DOI:** 10.1002/fsn3.72202

**Published:** 2026-08-10

**Authors:** Maroua Hadji, Khadidja Dehimi, Fethi Farouk Kebaili, Tahar Smaili, Toka Hadji, Larbi Derbak, Ilyas Yildiz, Fehmi Boufahja, Hamdi Bendif, Tarek H. Taha, Walid Elfalleh, Stefania Garzoli

**Affiliations:** ^1^ Biodiversity and Biotechnological Techniques for Plant Resources Valorization Laboratory (BTB_VRV), Department of Natural and Life Sciences, Faculty of Sciences University of M'sila, University Pole M'sila Algeria; ^2^ Department of Microbiology and Biochemistry, Faculty of Sciences University of M'sila, University Pole M'sila Algeria; ^3^ University Mohamed El Bachir El Ibrahimi of Bordj Bou Arreridj El Anasser Algeria; ^4^ Laboratory of Microbiological Engineering and Application, Biochemistry and Molecular and Cellular Biology Department, Faculty of Nature and Life Sciences University of Mentouri Brothers Constantine 1 Constantine Algeria; ^5^ Laboratory of the Development and Valorization of Plant Genetic Resources, Department of Biology and Plant Ecology University of Mentouri Brothers Constantine 1 Constantine Algeria; ^6^ Centre de Recherche en Technologies Agro‐Alimentaires Route de Targa Ouzemmour Bejaia Algeria; ^7^ Faculty of Health Sciences and Arts, Department of Molecular Biology and Genetics Tokat Gaziosmanpaşa University Tokat Turkey; ^8^ Department of Biology, College of Science Imam Mohammad Ibn Saud Islamic University (IMSIU) Riyadh Saudi Arabia; ^9^ Department of Chemistry and Technologies of Drug Sapienza University Rome Italy

**Keywords:** bioactivities, *Daucus carota* subsp. *maximus*, LC‐ESI‐MS/MS, natural compounds, pharmacokinetic potential

## Abstract

*Daucus carota* subsp. *maximus*, a biennial plant from the *Apiaceae* family, is valued for its aromatic properties. It is the largest wild carrot subspecies found in Algeria, characterized by its tall stem, triangular leaves, and large umbels. This study aimed to characterize the phenolic profile of organic extracts of *D. carota* subsp. *maximus* and evaluate their antioxidant, antibacterial, α‐amylase inhibitory, and ADME properties. The acetone extract contained the highest polyphenol content (305.17 ± 0.56 μg GAE/mg E), while the methanol extract had the highest flavonoid content (135.54 μg QE/mg E) and exhibited superior antioxidant activity across multiple assays through lower IC_50_ values in the DPPH (32.67 ± 0.74 μg/mL) and ABTS (13.76 ± 0.06 μg/mL) assays, and lower *A*_0.5_ values in the CUPRAC (47.79 ± 0.12 μg/mL), phenanthroline (131.65 ± 1.38 μg/mL), potassium ferricyanide (36.34 ± 0.83 μg/mL), and SNP (92.92 ± 0.73 μg/mL) assays. Using LC‐ESI‐MS/MS, a total of 19 phenolic compounds were detected, with rutin being the predominant one. The acetone extract exhibited marked antibacterial effects, especially against *Micrococcus luteus* and *Pseudomonas aeruginosa* , while the hexane extract achieved the greatest α‐amylase inhibitory activity, reaching 52.15% at 400 μg/mL. In silico ADME analysis suggested favorable absorption profiles for several compounds and possible BBB permeability for selected molecules. *D. carota* subsp. *maximus* exhibits significant bioactive properties, highlighting its potential applications as a source of antioxidant, antibacterial, and enzyme‐inhibitory agents. However, further in vivo investigations and formulation studies are required to validate these potential applications.

## Graphical abstract

Wild *Daucus carota* subsp. *Maximus:* chemical investigation and biological assessment.

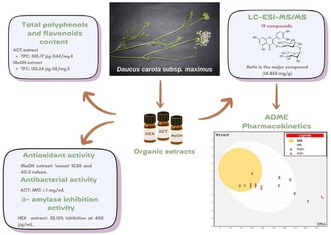

## 1 Introduction

In recent years, global interest in natural sources of bioactive compounds has increased due to their potential to improve health and provide therapeutic benefits, thereby attracting attention from the pharmaceutical, nutraceutical, and chemical industries. Wild aromatic and medicinal plants, traditionally utilized in herbal medicine and culinary practices, have become the focus of modern phytochemical research due to their diverse health promoting properties, including antioxidant, anti‐inflammatory, antimicrobial, and metabolic enzyme‐modulating activities (Twaij and Hasan 2022). Investigating the chemical composition and bioactivity of these plants can lead to the discovery of novel bioactive compounds with potential applications in both the development of functional foods that support human nutrition and health, and in pharmaceutical formulations such as supplements or therapeutic agents. Within this context, members of the *Apiaceae* family have gained considerable scientific attention due to their diverse phytochemical profiles. Among the genera within the *Apiaceae* family, *Daucus* stands out as one of the most recognized and extensively spread plants (Ayakodi et al. 2024). The genus comprises various species, with the wild carrot *Daucus carota* L., possibly being the most prominent. It is commonly named bird's nest or Queen Anne's lace, and it is characterized by its distinctive white, lacy flowers and feathery leaves. The plant produces a taproot that closely resembles the domesticated carrot ( *D. carota* subsp. *sativus*). However, unlike its domestic counterpart, the root of wild carrot is typically thin, hard, and strongly aromatic (Mitich 1996; Chizzola 2010). Plants of this species are valued for their anti‐inflammatory, antilithic, carminative, diuretic, antiseptic properties. They have been investigated for potential biological activities relevant to conditions such as cystitis, gout, prostatitis, and cancer (Ismail et al. 2023). Additionally, numerous studies have been conducted on wild carrot's bioactive compounds, revealing their pharmacological potential and adding to knowledge in fields ranging from herbal medicine to food applications (Zeinab et al. 2011; Staniszewska et al. 2005; Soković et al. 2009; Alves‐Silva et al. 2016; Gaglio et al. 2017).

*D. carota* subsp. *maximus* is among the wild plants studied for their interesting essential oil composition (Gaglio et al. 2017; Badalamenti et al. 2021; Silva et al. 2022). It belongs to the *Apiaceae* family as a biennial plant that blooms during the months of May through August. The stem can reach a length of 2 m completely bristling with stiff hairs and is either single or flanked by basal oblique shoots. Also, it has triangular‐shaped leaves and large umbels, and it has a white color and spherical shape once fully flowered. Compared to other wild plant subspecies in Algeria, *D. carota* subsp. *maximus* is the largest wild carrot subspecies found in the country (Tani et al. 2021). Previous phytochemical studies on *D. carota* subsp. *maximus* have primarily focused on its essential oil properties (Gaglio et al. 2017; Badalamenti et al. 2021; Silva et al. 2022). To date, scientific literature on the phenolic profile of *D. carota* subsp. *maximus* remains highly limited, with only a single study evaluating the phenolic profile of the aqueous extract and assessing its antioxidant activity (Hadji et al. 2024). Consequently, the chemical composition and biological potential of its non‐volatile organic extracts remain poorly explored. Because different classes of phytoconstituents require extraction across a spectrum of solvent polarities, a single solvent approach leaves a significant gap in our understanding of the full profile of plants. To address this gap, the present study comprehensively investigates the bioactive constituents of the aerial parts of *D. carota* subsp. *maximus* and evaluates their biological and pharmacokinetic relevance. This investigation is based on the premise that the use of solvents with different polarities enables the extraction of diverse classes of phytochemicals with potentially distinct bioactivities. Indeed, previous literature confirms significant correlations between solvent polarity, phytochemical composition, and biological outcomes like antioxidant, antimicrobial, and enzyme inhibitory effects (Hadji et al. 2025). Sequentially employing *n*‐hexane, acetone, and methanol, we provide the first detailed phenolic profiling of the organic extracts of this subspecies via LC‐ESI‐MS/MS, alongside total polyphenol and flavonoid quantification. Furthermore, the resulting extracts are evaluated for their antioxidant, antibacterial, and α‐amylase inhibitory activities, paired with in silico ADME profiling, to firmly establish the relationship between the chemical diversity of the plant and its functional, bioavailable profile.

## 2 Materials and Methods

### 2.1 Chemical Materials and Spectral Measurements

All required chemical reagents were obtained from Sigma‐Aldrich (Taufkirchen, Germany), including Folin Ciocalteu phenol reagent, sodium carbonate (Na_2_CO_3_), aluminum chloride (AlCl_3_), DPPH (2,2‐diphenyl‐1‐picrylhydrazyl), ABTS (2,2′‐azinobis (3‐ethylbenzoline‐6‐sulfonic acid) diammonium salt), potassium persulfate (K_2_S_2_O_8_), neocuproine (NC) (2,9‐dimethyl‐1,10‐phenanthroline), ammonium acetate (ACNH_4_), copper chloride dihydrate (CuCl_2_·2H_2_O), dipotassium phosphate (K_2_HPO_4_), monopotassium phosphate (KH_2_PO_4_), potassium ferricyanide (K_3_Fe(CN)_6_), trichloroacetic acid (TCA), ferric chloride (FeCl_3_), phenanthroline, silver, quercetin, butylated hydroxyanisole, α‐tocopherol, Trolox, α‐amylase, starch, hydrochloric acid (HCl), sodium chloride (NaCl), acarbose. Also, the used solvents were sourced from Sigma‐Aldrich and Honeywell, including *n*‐hexane, acetone, methanol, dimethylsulfoxide (DMSO). For LC‐ESI‐MS/MS analysis, the phenolic reference standards were obtained from Sigma‐Aldrich (Merck, Germany) with purities of ≥ 95%. These included *p*rotocatechuic acid, *catechin*, chlorogenic acid, hydroxybenzaldehyde, vanillic acid, caffeic acid, vanillin, salicylic acid, p‐coumaric acid, polydatin, resveratrol, trans‐ferulic acid, isoquercitrin, rutin, hesperidin, kaempferol‐3‐glucoside, fisetin, quercetin, kaempferol. The antibiotics used in the antibacterial assays included vancomycin (VN), ciprofloxacin (CIP), cefalexin (CN), imipenem (IMP), and G: gentamicin, which were obtained as commercial standard discs from Oxoid (Thermo Fisher Scientific, UK). On the other hand, UV spectra absorbances were measured using a Multiskan SkyHigh Microplate spectrophotometer from Thermo Scientific.

### 2.2 Plant Material Collection and Identification

The aerial parts of *D. carota* subsp. *maximus*, growing wild in Algeria, were gathered from the M'sila region (35°42′ N, 4°33′ E) in June 2021 during the flowering stage. Plant material was collected in the absence of specific local regulations governing the harvesting of wild plant species in the study area. The investigated species is not classified as a protected plant; therefore, no formal collection permit was required for sample collection. A number of individual plants were randomly collected and pooled to obtain a representative bulk sample for subsequent analyses. The species was taxonomically identified by Pr. Smaili T., Faculty of Sciences, Department of Life and Nature Sciences, Mohamed Boudiaf University, M'sila, Algeria, based on the Flora of Algeria (Quezel and Santa 1963) and Tani et al. (2021). A voucher specimen (MH‐01) was prepared and deposited in the departmental herbarium, where it is publicly accessible upon request.

### 2.3 Bioactive Compounds Extraction

To ensure an effective extraction of active compounds from *D. carota* subsp. *maximus*, the collected aerial parts were thoroughly washed, air‐dried in the absence of direct sunlight under ambient temperature conditions (≤ 30°C), and finely ground using a laboratory mill. A sequential extraction procedure was then applied using organic solvents of increasing polarity (*n*‐hexane, acetone, and methanol) to obtain a broad range of phytochemicals, following the method described by Hadji et al. (2025). First, 50 g of plant material powder was soaked with 500 mL of *n*‐hexane for 48 h. After filtration, the plant residue was subjected to a second extraction with 500 mL of acetone. The same residue obtained after the second filtration was then further extracted with 500 mL of methanol. Subsequently, after each extraction step, solvents were removed using a rotary evaporator at 40°C, and the resulting extracts were then oven‐dried at 40°C until constant weight to ensure complete removal of residual solvents before storage. The dried extracts (hexane: HEX, acetone: ACT, methanol: MeOH) were stored in amber vials at 4°C until analysis. All extractions were conducted at ambient temperature, without exposure to light and under occasional stirring.

### 2.4 Total Phenolic Compound Estimation

#### 2.4.1 Total Polyphenol Content

Total phenolic content was determined using a modified Folin–Ciocalteu colorimetric method adapted from Derbak et al. (2024). This assay is based on the reduction of the Folin–Ciocalteu reagent by phenolic constituents, resulting in the formation of a blue chromophore detectable by spectrophotometry. 20 μL of each extract solution (1 mg/mL) was combined with 100 μL of Folin–Ciocalteu reagent (10%, v/v) in a 96‐well microplate. After an initial incubation period of 4 min, 80 μL of sodium carbonate solution (7.5%, w/v) was added to the mixture. The reaction was subsequently maintained for 2 h at room temperature under dark conditions. Absorbance was recorded at 765 nm using a microplate reader. Quantification was performed using a gallic acid calibration curve constructed over the concentration range of 0–200 μg/mL, with the following regression equation: *y* = 0.0043*x* + 0.0363 (*R* ^2^ = 0.991). All absorbance values were corrected using a reagent blank. Measurements were performed in triplicate, and results were expressed as micrograms of gallic acid equivalents per milligram of extract (μg GAE/mg E).

#### 2.4.2 Total Flavonoids Content

Total flavonoid content was quantified using a modified aluminum chloride colorimetric method adapted from Shraim et al. (2021). The assay is based on the formation of stable complexes between flavonoids and aluminum ions, producing a measurable chromogenic response. A volume of 100 μL of extract solution (1 mg/mL) was combined with 100 μL of aluminum chloride solution (2%, w/v) in a microplate. The mixture was incubated for 15 min at room temperature prior to absorbance measurement at 430 nm using a microplate reader. Quantification was achieved using a quercetin calibration curve in the range of 0–200 μg/mL, with the regression equation: *y* = 0.0049*x* + 0.015 (*R* ^2^ = 0.996). Absorbance values were corrected against a reagent blank, and all analyses were performed in triplicate. Results were expressed as micrograms of quercetin equivalents per milligram of extract (μg QE/mg E).

### 2.5 LC‐ESI‐MS/MS Study

The LC‐ESI‐MS/MS technique was employed to analyze the phytochemical profile of *D. carota* subsp. *maximus* organic extracts. Separation was performed using a Poroshell 120 EC‐C18 column (Agilent Technologies, 100 mm × 3.0 mm, 2.7 μm particle size) under reversed‐phase chromatography conditions. Phenolic compounds were analyzed using an Agilent 1260 Infinity II liquid chromatography system coupled to a tandem mass spectrometer (Erenler et al. 2023).

For sample preparation, 50 mg of each extract was dissolved in 1 mL of methanol. Subsequently, 1 mL of hexane was added to remove nonpolar interferences and improve phase separation. The mixture was centrifuged for 10 min at 9000 rpm, and 100 μL of the methanolic phase was carefully collected. This phase was then diluted with a water/methanol mixture (450/450 μL), filtered through a 0.22 μm membrane, and injected into the LC–MS/MS system (injection volume: 5.12 μL; flow rate: 0.40 mL/min).

The mobile phase consisted of water (A) containing 0.1% formic acid and 5.0 mM ammonium formate, and methanol (B) containing the same additives. The gradient program for solvent B was set as follows: 25% (1–3 min), 50% (4–12 min), 90% (13–21 min), and 3% (22–25 min). The column temperature was maintained at 40°C. The capillary voltage was 4000 V, nebulizing gas (N_2_) flow rate was 11 L/min, pressure was 15 psi, and gas temperature was 300°C. The mass spectrometer was equipped with an electrospray ionization (ESI) source and operated in both positive and negative ionization modes according to the characteristics of the target analytes. Data acquisition was performed in multiple reaction monitoring (MRM) mode using precursor‐to‐product ion transitions for each compound. Method validation parameters, including linearity range, limit of detection (LOD), and limit of quantification (LOQ), were determined according to Yilmaz (2020). The validation was performed by assessing the sensitivity and linearity of each target analyte under optimized LC–MS/MS conditions. LOD and LOQ were determined based on serial dilutions of standard solutions, where the lowest concentration providing a signal‐to‐noise ratio (*S*/*N*) of approximately 3:1 was defined as the LOD, and a ratio of 10:1 was defined as the LOQ, according to the following expressions: LOD = *S*/*N* = 3, LOQ = *S*/*N* = 10.

Linearity was evaluated using calibration curves constructed at eight concentration levels, each analyzed in triplicate. Calibration plots were generated by plotting the ratio of analyte concentration to internal standard concentration against the corresponding ratio of analyte peak area to internal standard peak area. Excellent linearity was observed for all compounds, with correlation coefficients (*R* ^2^) greater than 0.99 across the studied concentration ranges. Detailed information on ion transitions, ionization modes, linear ranges, LOD, LOQ, and *R* ^2^ values is provided in Table 2.

### 2.6 In Vitro Antioxidant Activities

The antioxidant potential of *D. carota* subsp. *maximus* extracts was assessed through various spectrophotometric assays, including radical scavenging assays and antioxidant reducing power assays. For comparison, five antioxidant standards were employed: ascorbic acid (AsA), butylated hydroxyanisole (BHA), quercetin (QCT), trolox (TX), and α‐tocopherol (TCP).

#### 2.6.1 DPPH Radical Scavenging Assay

The free radical scavenging activity of *D. carota* subsp. *maximus* extracts was evaluated using the DPPH assay, following the method described by Sali̇hoğlu et al. (2022) with slight modifications. This assay is based on the reduction of the stable DPPH radical, which results in a decrease in absorbance. 40 μL of extract or standard solution at various concentrations was mixed with 160 μL of DPPH solution (60 μM) in a microplate. The reaction mixture was incubated for 30 min at room temperature in the dark. After incubation, the absorbance was measured at 517 nm using a microplate reader. The radical scavenging activity was calculated as a percentage of DPPH inhibition according to the following equation: $\text{DPPH scavenging activity}\%=\frac{\left(A_{\text{control}}-A_{\text{sample}}\right)\;}{A_{\text{control}}}$×100, in which *A*_control_ is the absorbance of DPPH solution with extract or standard solvent, and *A*_sample_ is the absorbance of the extract or standards with DPPH solution. QCT, BHA and TCP were used as reference antioxidant standards for comparison.

#### 2.6.2 ABTS Cation Radical Scavenging Assay

The ABTS radical cation scavenging activity of *D. carota* subsp. *maximus* extracts was determined using a modified version of the method described by Re et al. (1999) and by Hussen and Endalew (2023). This assay is based on the ability of antioxidants to quench the ABTS^+^ radical, resulting in a decrease in absorbance. The ABTS^+^ radical solution was prepared by mixing 7 mM ABTS aqueous solution with 2.45 mM potassium persulfate (K_2_S_2_O_8_). The mixture was allowed to react in the dark for 12–16 h at room temperature to generate the radical cation. Prior to use, the solution was diluted with ethanol to obtain an absorbance of 0.70 at 734 nm. For the assay, 40 μL of extract or standard solution at different concentrations was added to 160 μL of the prepared ABTS^+^ solution in a microplate. After incubation for 10 min at room temperature, the absorbance was measured at 734 nm using a microplate reader. The percentage of ABTS^+^ radical scavenging activity was calculated using the following equation: $\text{ABTS scavenging activity}\%=\frac{\left(A_{\text{control}}-A_{\text{sample}}\right)\;}{A_{\text{control}}}\times 100$; in which *A*_control_ is the absorbance of ABTS solution with extract or standard solvent, and *A*_sample_ is the absorbance of the extract or standard with ABTS solution. The results were expressed as a percentage of half maximum inhibitory concentration (IC_50_ in μg/mL), which is the concentration required for 50% inhibition of ABTS radical. QCT and BHA were used as reference antioxidant standards for comparison.

#### 2.6.3 Cupric Reducing Antioxidant Capacity (CUPRAC)

The cupric ion reducing antioxidant capacity of *D. carota* subsp. *maximus* extracts was evaluated using the CUPRAC method described by Apak et al. (2004) and İpek et al. (2024) with minor modifications. This assay is based on the reduction of Cu^2+^ to Cu^+^ by antioxidant compounds in the presence of neocuproine, forming a colored complex measurable spectrophotometrically. In a 96‐well microplate, 40 μL of extract or standard solution at various concentrations was mixed with 50 μL of copper (II) chloride dihydrate solution (10 mM), 50 μL of neocuproine solution (7 mM), and 60 μL of ammonium acetate buffer (1 M, pH 7.0). The reaction mixture was incubated for 60 min at room temperature. Subsequently, the absorbance was recorded at 450 nm using a microplate reader. The antioxidant capacity was expressed as A_0.5_ (μg/mL), defined as the concentration of the sample required to reach an absorbance of 0.50 under the assay conditions. QCT and TCP were used as reference antioxidant standards for comparison.

#### 2.6.4 Potassium Ferricyanide Reducing Power Assay

The ferric ion reducing ability of *D. carota* subsp. *maximus* extracts was assessed using a method based on Oyaizu (1986) with modifications described by Dabbou et al. (2015). This assay evaluates the capacity of antioxidants to reduce Fe^3+^ to Fe^2+^, leading to the formation of a colored complex measurable spectrophotometrically. In a 96‐well microplate, 10 μL of extract or standard solution at various concentrations was mixed with 40 μL of phosphate buffer (pH 6.6) and 50 μL of potassium ferricyanide solution (1%, w/v). The mixture was incubated at 50°C for 20 min. Following incubation, 50 μL of trichloroacetic acid (10%) was added, followed by 40 μL of distilled water and 10 μL of ferric chloride solution (0.1%, w/v). The absorbance of the resulting solution was measured at 700 nm using a microplate reader. The antioxidant capacity was expressed as A_0.5_ (μg/mL), defined as the concentration required to reach an absorbance of 0.50 under the assay conditions. QCT, BHA, and TCP were used as reference antioxidant standards for comparison.

#### 2.6.5 Phenanthroline Reducing Power Assay

The ferric ion reducing activity of *D. carota* subsp. *maximus* extracts was evaluated using a modified phenanthroline assay, based on the method described by Loucif et al. (2021) with some modifications. This assay measures the ability of antioxidants to reduce Fe^3+^ to Fe^2+^, forming a colored Fe^2+^–phenanthroline complex detectable spectrophotometrically. 10 μL of extract or standard solution at varying concentrations was combined with 50 μL of ferric chloride solution (0.2%, w/v), 30 μL of phenanthroline solution (0.5%, w/v), and 110 μL of methanol in a microplate. The mixture was incubated in the dark at 30°C for 20 min, after which the absorbance was measured at 510 nm using a microplate reader. The antioxidant activity was expressed as *A*_0.5_ (μg/mL), defined as the concentration required to reach an absorbance of 0.50 under the assay conditions.

#### 2.6.6 Silver Nanoparticles (SNP) Assay

The capacity of *D. carota* subsp. *maximus* extracts to reduce silver ions (Ag^+^) to elemental silver, resulting in the formation of silver nanoparticles (AgNPs), was evaluated using a modified method based on Özyürek et al. (2012). This assay assesses the antioxidant‐driven reduction of silver ions, which can be monitored spectrophotometrically. 20 μL of extract solution at varying concentrations was mixed with 130 μL of silver nitrate solution (1.0 mM) and 50 μL of distilled water in a microplate. The reaction mixture was incubated at 25°C for 30 min. Following incubation, the formation of silver nanoparticles was monitored by measuring the absorbance at 423 nm using a microplate reader. The antioxidant activity was expressed as A_0.5_ (μg/mL), defined as the concentration of extract required to achieve 50% of maximal silver ion reduction under the assay conditions. TX and AsA were used as reference antioxidant standards for comparison.

### 2.7 Antibacterial Activity

#### 2.7.1 Bacterial Strains

The antibacterial activity of *D. carota* subsp. *maximus* extracts was evaluated against standard reference strains obtained from the American Type Culture Collection (ATCC) and supplied by the microbiology laboratory of Mohamed Boudiaf University, M'sila, Algeria. All tested strains are known for their pathogenic potential. The Gram‐negative bacteria included *Escherichia coli* ATCC 25922, *Pseudomonas aeruginosa* ATCC 27853, and *Enterobacter cloacae* ATCC 13047, whereas the Gram‐positive strains comprised *Staphylococcus aureus* ATCC 25923, *Micrococcus luteus* ATCC 4698, and *Bacillus subtilis* ATCC 9372.

#### 2.7.2 Preparation of Inoculum

The bacterial strains were maintained active by transplanting them for 18 h at 37°C on a nutrient agar that is conducive to their growth. Each bacterial colony was picked and added to 9 mL of physiological saline solution in a sterile tube. After mixing, the inoculum was adjusted between 0.08 and 0.13 optical densities at a wavelength of 620 nm. The inoculum final concentration is approximately between 10^7^ and 10^8^ CFU/mL (Javed et al. 2023).

#### 2.7.3 Antibacterial Susceptibility

The evaluation of the antibacterial susceptibility of *D. carota* subsp. *maximus* organic extracts was executed using the method of agar well diffusion, which is extensively utilized due to its simplicity and efficacy in determining the antibacterial activity (Damtie and Mekonnen 2020). Firstly, Petri dishes were filled with 20 mL of sterile Mueller‐Hinton agar medium. After it solidified, a microbial suspension with an optical density of 10^8^ CFU/mL was distributed on its surface. Accordingly, three 8 mm wells were created in the nutrient agar plates to assess antibacterial activity. Subsequently, extracts were dissolved using 5% dimethyl sulfoxide (DMSO); then, 25 μL of each extract (200 mg/mL) was added to each well on Petri dishes using a micropipette. The Petri dishes were kept at 4°C for 1 h. After that, they were incubated at 37°C for 24 h. Ciprofloxacin, vancomycin, cephalexin, and imipenem served as positive controls for the experiment. The 5% DMSO solution was tested as the negative control under the same experimental conditions as the extracts. The diameter of the inhibition zone surrounding each well served as an indicator for antibacterial potential. The experiments were performed in triplicate.

#### 2.7.4 Minimum Inhibitory Concentration (MIC) and Minimum Bactericidal (MBC) Concentration

The minimum inhibitory concentration (MIC) and minimum bactericidal concentration (MBC) were determined using the broth microdilution method in 96‐well microplates, as described in by Damtie and Mekonnen (2020) with some modifications. On nutrient agar, 18 h‐young cultures of bacteria were used to make bacterial suspensions, which were then measured to a standard turbidity of 0.5 McFarland. In 5% DMSO, the extracts were first dissolved. The microdilution method was used on a 96‐well microplate to calculate the MIC values for extracts against several bacterial strains. Specifically, 90 μL of nutrient broth and 10 μL of inoculum were added into each well of 96‐well microplates. The stock solution of extracts was put in the initial wells in a volume of 100 μL. Following that, 100 μL of extracts' dilution was added to seven successive wells. The final well was used as a growth control and contained 10 μL of inoculum and 190 μL of nutritional broth without extracts. The final volumes in each well were 200 μL, and the final concentrations ranged from 200 to 0.78125 mg/mL. Gentamicin was used as a positive control with concentrations ranged from 1.25 to 0.019 mg/mL. After that, the microplates were incubated for 24 h at 37°C. MICs were identified as the lowest concentrations at which no visible bacterial growth was observed. For MBC determination, spots from MIC well microplates were transferred onto sterile agar plates and incubated for 18 h at 37°C. MBC was defined as the lowest concentration that showed no colony growth on agar after incubation.

### 2.8 In Vitro α‐Amylase Inhibition Activity

The α‐amylase inhibitory activity of *D. carota* subsp. *maximus* extracts was assessed using a modified Lugol's iodine method, based on Quan et al. (2019). Acarbose was used as the reference standard. This assay evaluates the ability of the extracts to inhibit the enzymatic hydrolysis of starch.

Stock solutions of the extracts were prepared at 4 mg/mL. For the assay, 25 μL of extract solution was mixed with 50 μL of α‐amylase solution (1 unit in phosphate buffer, pH 6.9, containing 6 mM NaCl) and incubated at 37°C for 10 min. Following this pre‐incubation, 50 μL of 0.1% starch solution was added, and the mixture was further incubated at 37°C for an additional 10 min. The reaction was terminated by adding 25 μL of 1 mM HCl, followed by the addition of 100 μL of Lugol's iodine solution (5 mM). Absorbance was measured at 630 nm using a microplate reader.

The percentage of α‐amylase inhibition was calculated using the following equation: $\text{alpha amylase inhibition}\%=\frac{\left(A_{s}-A_{e}\right)}{\left(A_{c}-A_{e}\right)}\times 100$; where *A*_s_ is the absorbance of the reaction mixture containing enzyme and plant extract, *A*_e_ is the absorbance of the reaction mixture containing enzyme and without plant extract, and *A*_c_ is the absorbance of the reaction mixture without enzyme. Inhibitory activity was expressed as IC_50_ (μg/mL), defined as the concentration of extract required to inhibit 50% of the α‐amylase activity under the assay conditions.

### 2.9 Bioavailability and Pharmacokinetic Characteristics

The bioavailability of the phytochemicals identified in *D. carota* subsp. *maximus* organic extracts was estimated through computational approaches considering essential physicochemical features, including molecular weight, lipophilicity, polarity, and aqueous solubility, as outlined by Hchicha et al. (2021). In addition, their pharmacokinetic behavior was predicted using the SwissADME web platform (http://www.swissadme.ch, accessed on November 15, 2023), which was applied to generate ADME profiles related to absorption, distribution, metabolism, and excretion. Canonical SMILES of each compound were used as input for all calculations. These in silico evaluations were used to provide an initial assessment of the drug likeness and pharmacokinetic potential of the identified compounds.

### 2.10 Statistical Analysis

The results were reported as the mean ± SD of three technical replicates. All IC_50_ and *A*_0.5_ results were determined using linear regression analysis. Statistical analysis was performed using the XLSTAT 2016 V1.0 statistical package (XLSTAT Addinsoft Inc., New York, NY, USA). Group differences were evaluated using one‐way analysis of variance (ANOVA) followed by Tukey's honestly significant difference (HSD) test for multiple comparisons. Statistical significance was determined at *p* < 0.05.

## 3 Results and Discussion

### 3.1 Extraction Yield, Total Polyphenols, and Flavonoids Content

The extraction yield, total polyphenols, and total flavonoids content were quantified for all *D. carota* subsp. *maximus* organic extracts. The corresponding data are presented in Table 1. In accordance with the results, all solvents demonstrate extraction efficiency that ranges from 2.24% to 9.02%. The higher extraction rate was found in MeOH extract which reached to 9.02%, followed by ACT extract and HEX extract with a slightly similar rate that is estimated at 2.47% and 2.24%, respectively, with no significant difference at *p* < 0.05. As indicated in Table 1. ACT extract presents the highest TPC compared to other extracts, which was approximately 305.17 ± 0.56 μg GAE/mg E. Followed by MeOH extract with TPC of 264.95 ± 0.71 μg GAE/mg E. Concerning TFC, MeOH extract showed the best content in flavonoids, with concentration of 135.54 ± 0.59 μg QE/mg E. ACT extract demonstrated a TFC estimated value of 76.35 ± 0.51 μg QE/mg E. Meanwhile, HEX extract exhibited the lowest polyphenols, and flavonoids content, with measurements of 34.88 ± 0.23 μg GAE/mg E, and 27.36 ± 0.70 μg QE/mg E, respectively.

**TABLE 1:** Yield, total polyphenols, and flavonoids contents of *Daucus carota* subsp. *maximus* organic extracts.

Extracts	Yield (%)	TPC (μg GAE/mg E)	TFC (μg QE/mg E)
HEX	2.24 ± 0.43^(a)^	34.88 ± 0.23^(a)^	27.36 ± 0.70^(c)^
ACT	2.47 ± 0.12^(a)^	305.17 ± 0.56^(c)^	76.35 ± 0.51^(b)^
MeOH	9.02 ± 0.82^(b)^	264.95 ± 0.71^(b)^	135.54 ± 0.59^(a)^

*Note:* Groups marked with different letters represent values that differ significantly from each other.

The extraction yield, total polyphenols, and flavonoids content of *D. carota* subsp. *maximus* organic extracts, significantly impacted by extraction solvent polarity. According to Nakilcioğlu‐Taş and Ötleş (2021), the polarity and properties of the solvent influence both phytochemical composition and extraction yield. The higher yield observed may be attributed to the greater polarity of the solvent, since it would enhance the solubility of an extensive variety of phenolic substances (Muhamad et al. 2014). The results of TPC suggest that acetone is an effective solvent for the extraction of polyphenols from *D. carota* subsp. *maximus*, followed by methanol. Acetone has a moderate polarity in comparison to non‐polar solvents like hexane, and strong polar solvents like water (Abubakar and Haque 2020). This moderate polarity is due to the presence of polar carbonyl group (CO) and two non‐polar methyl groups (CH_3_), which make it more effective in dissolving a wider range of polar and moderately polar compounds from plant materials, including phenolic compounds.

The results of TFC suggest that methanol was a better solvent for the extraction of flavonoids from *D. carota* subsp. *maximus*. It is acknowledged in the literature that flavonoids are highly soluble in polar solvents like methanol (Ferreira and Pinho 2012). However, it should be noted that, due to the sequential extraction procedure, the phytochemical content of each fraction may be influenced by the progressive removal of compounds during the preceding extraction steps. Only a few studies focus on phytochemicals extraction from *Daucus* species using different‐polarity solvents. Nabi et al. (2023) revealed that the highest TPC in *D. carota* L. seeds extracts was found in the methanolic extract, while the lowest content was found in the *n*‐hexane extract. Furthermore, flavonoid content was similar and higher in both polar (methanol) and nonpolar (*n*‐hexane) solvents. Compared with the aqueous extract of *D. carota* subsp. *maximus* reported by Hadji et al. (2025), which showed a total extraction yield of 16.37% ± 1.43% with TPC and TFC values of 99.00 ± 0.71 μg GAE/mg E and 43.06 ± 0.77 μg QE/mg E, respectively, the present study demonstrated higher polyphenolic and flavonoid contents in methanol and acetone extracts, although with a lower extraction yield than the previously reported aqueous extract. This suggests that solvent selection significantly affects both extraction yield and phytochemical composition, which may subsequently influence bioactivities.

### 3.2 LC‐ESI‐MS/MS Profiling

**FIGURE 1:**
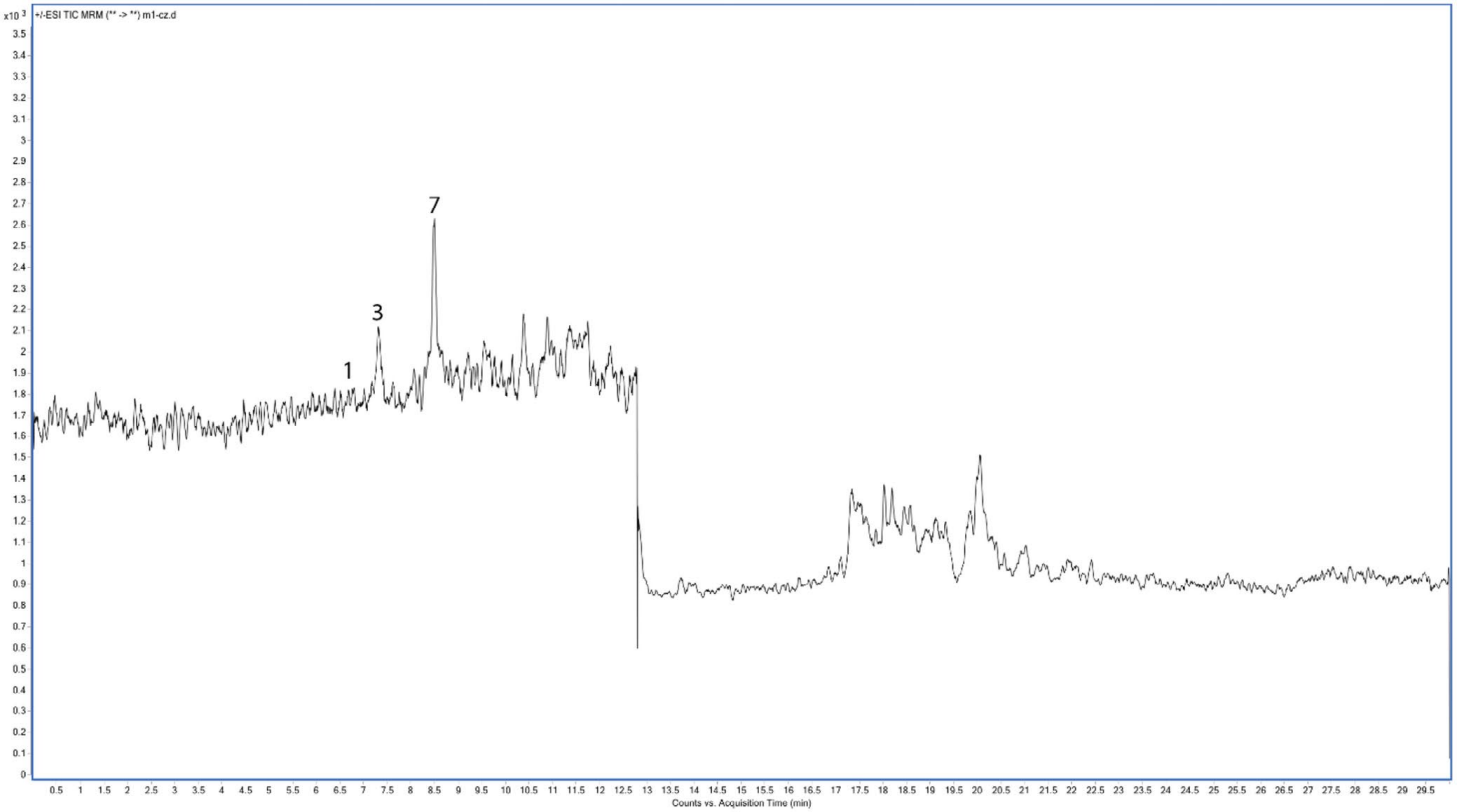
LC‐ESI‐MS/MS chromatogram of *Daucus carota* subsp. *maximus* hexanic extract: (1) protocatechuic acid, (3) chlorogenic acid, (7) vanillin.

**FIGURE 2:**
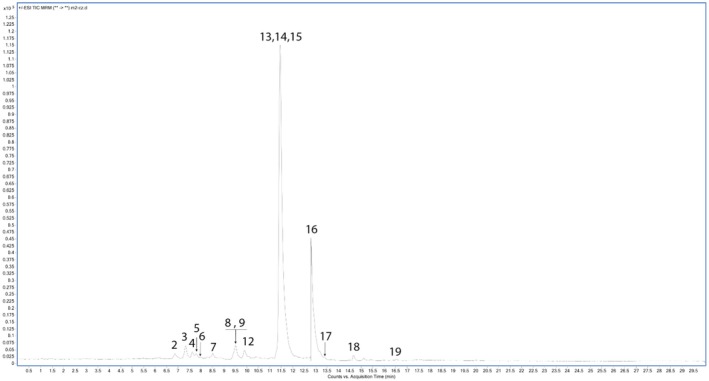
LC‐ESI‐MS/MS chromatogram of *Daucus carota* subsp. *maximus* acetonic extract: (2) catechin, (3) chlorogenic acid, (4) hydroxybenzaldehyde, (5) vanillic acid, (6) caffeic acid, (7) vanillin, (8) salicylic acid, (9) p‐coumaric acid, (12) trans‐ferulic acid, (13) isoquercitrin, (14) rutin, (15) hesperidin, (16) kaempferol‐3‐glucoside, (17) fisetin, (18) quercetin, (19) kaempferol.

**FIGURE 3:**
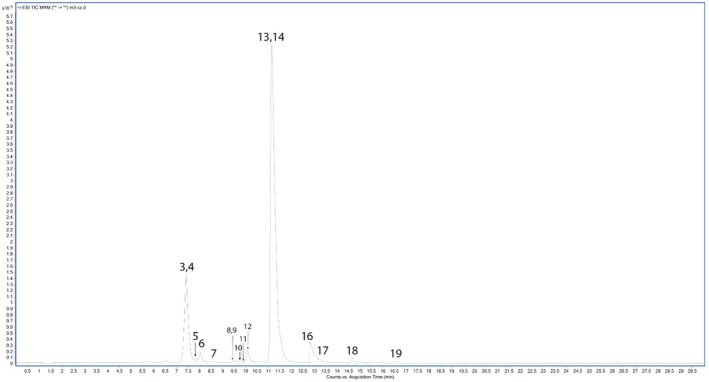
LC‐ESI‐MS/MS chromatogram of *Daucus carota* subsp. *maximus* methanolic extract: (3) Chlorogenic acid, (4) hydroxybenzaldehyde, (5) vanillic acid, (6) caffeic acid, (7) vanillin, (8) salicylic acid, (9) p‐coumaric acid, (10) polydatin, (11) resveratrol, (12) trans‐ferulic acid, (13) isoquercitrin, (14) rutin, (16) kaempferol‐3‐glucoside, (17) fisetin, (18) quercetin, (19) kaempferol.

The analysis of HEX, ACT, and MeOH extracts isolated from *D. carota* subsp. *maximus* aerial parts was performed using liquid chromatography coupled with tandem mass spectrometry. Nineteen phenolic compounds were identified based on their LC‐ESI‐MS/MS precursor‐to‐product ion transitions, MS/MS fragmentation data provided by the analytical method, and retention times. The LC‐ESI‐MS/MS chromatograms of *D. carota* subsp. *maximus* hexanic extract, acetonic extract, and methanolic extract were reported in Figures 1, 2, and 3, respectively. The identified compounds were quantified and listed in Table 2. These compounds included seven phenolic acids: protocatechuic acid, vanillic acid, salicylic acid, caffeic acid, p‐coumaric acid, trans‐ferulic acid, and chlorogenic acid; two phenolic aldehydes: hydroxybenzaldehyde and vanillin; two stilbenoids: polydatin and resveratrol; and eight flavonoids: catechin, isoquercitrin, rutin, hesperidin, kaempferol‐3‐glucoside, fisetin, quercetin, kaempferol. The major phenolic compound was rutin in MeOH extract, with the highest concentration estimated at 14.856 mg/g, followed by chlorogenic acid in the same extract with a concentration of 7.764 mg/g. Subsequently, the amount of hesperidin present in ACT extract was 5.447 mg/g, and the amount of isoquercitrin detected in MeOH was 2.217 mg/g. The rutin present in ACT extract showed a concentration of 1.849 mg/g. The rest of the identified compounds were present in different concentrations that did not exceed 1 mg/g. Furthermore, MeOH extract shows the highest concentration of identified phenolic compounds, reaching 26.081 mg/g, followed by ACT extract at 8.809 mg/g. In contrast, HEX extract presents the lowest concentration at 0.110 mg/g.

**TABLE 2:** Phenolic compounds detected and quantified using LC‐ESI‐MS/MS in *Daucus carota* subsp. *maximus* extracts.

No.	Compound	RT (min)	Concentration (mg/g)			Ion source	Ion mod	Ion transitions	Linearity range (μg/L)	*R* ^2^	LOQ (μg/L)	LOD (μg/L)
			HEX	ACT	MeOH							
1	Protocatechuic acid	6.077	0.090	ND	ND	ESI	Neg.	153.0 → 109.0	15.627–250	0.9969	131.731	31.567
2	Catechin	6.849	ND	0.001	ND	ESI	Neg.	288.9 → 245.1	343.752–5500	0.9946	1705.501	750.130
3	Chlorogenic acid	7.326	0.012	0.187	7.764	ESI	Neg.	353.0 → 191.0	31.27–500	0.9981	259.023	115.860
4	Hydroxybenzaldeyde	7.618	ND	0.024	0.012	ESI	Neg.	121.0 → 92.0	15.625–250	0.9993	128.653	49.744
5	Vanillic acid	7.750	ND	0.241	0.230	ESI	Neg.	167.0 → 151.8	1250–20,000	0.9958	4242.134	2190.422
6	Caffeic acid	7.828	ND	0.028	0.079	ESI	Neg.	178.9 → 135.1	31.25–500	0.9994	241.163	69.206
7	Vanillin	8.506	0.008	0.012	0.009	ESI	Pos.	153.0 → 125.0	62.6–1000	0.9949	405.412	145.887
8	Salicylic acid	9.503	ND	0.062	0.286	ESI	Neg.	137.0 → 93.1	112.5–1800	0.9981	829.643	476.697
9	p‐coumaric acid	9.581	ND	0.029	0.043	ESI	Neg.	163.0 → 119.0	31.25–500	0.9987	175.419	35.349
10	Polydatin	9.764	ND	ND	0.002	ESI	Pos.	390.9 → 228.9	7.8126–125	0.9987	18.422	11.477
11	Resveratrol	9.843	ND	ND	0.003	ESI	Pos.	229.0 → 107.0	18.78–300	0.991	135.588	45.810
12	trans‐ferulic acid	10.088	ND	0.237	0.537	ESI	Neg.	193.1 → 133.9	31.29–1000	0.995	115.289	61.195
13	Isoquercitrin	11.461	ND	0.549	2.217	ESI	Pos.	464.9 → 302.8	18.95–300	0.9982	112.770	19.388
14	Rutin	11.687	ND	1.849	14.856	ESI	Pos.	611.0 → 302.8	124–2000	0.998	595.599	240.677
15	Hesperidin	11.695	ND	5.447	ND	ESI	Pos.	611.0 → 302.9	32.15–500	0.9957	166.499	33.007
16	Kaempferol‐3‐glucoside	12.827	ND	0.122	0.002	ESI	Pos.	448.8 → 286.9	7.8133–124	0.9997	45.239	11.610
17	Fisetin	13.420	ND	0.002	0.022	ESI	Pos.	287.0 → 137.0	15.622–250	0.9954	108.962	44.366
18	Quercetin	14.799	ND	0.006	0.006	ESI	Neg.	300.8 → 151.0	27.4–440	0.9964	169.129	46.555
19	Kaempferol	16.546	ND	0.012	0.013	ESI	Neg.	284.9 → 116.9	311.5–10,000	0.9997	1868.3	540.04
Total quantification (mg/g)			0.110	8.809	26.081							

Abbreviations: ND, not detected; Neg, negative; Pos, positive; RT, retention time.

Variations in the quantity and type of compounds detected in the extracts may result from the differing polarities of the solvents used, as solvent polarity influences compound solubility (Muhamad et al. 2014). Specifically, the extracting solvent can only dissolve and extract compounds that have similar polarity (Chew et al. 2011).

Based in Table 2, two compounds were detected in all extracts, chlorogenic acid and vanillin. Vanillin, which is extracted from vanilla, and chlorogenic acid, which is abundantly present in coffee and several fruits, both have many beneficial health effects. These substances are well‐known for having strong anti‐inflammatory and antioxidant properties, which support their capacity to reduce oxidative stress and inflammation in the body while enhancing overall wellness (La Rosa et al. 2023; Arya et al. 2021). Importantly, chlorogenic acid was particularly abundant in the MeOH extract (7.764 mg/g), which is consistent with the significantly higher antioxidant activity observed in this extract compared to ACT and HEX. This suggests that chlorogenic acid may contribute to the radical scavenging capacity of the methanolic extract, supported by the well documented ability of chlorogenic acid to act as an efficient radical scavenger and reducing agent (La Rosa et al. 2023).

Quercetin, isoquercitrin, kaempferol, kaempferol‐3‐glucoside, fisetin, hydroxybenzaldehyde, trans‐ferulic acid, p‐coumaric acid, caffeic acid, salicylic acid and vanillic acid, are commonly found in fruits, vegetables, and medicinal plants and have been reported in the literature to exhibit a range of biological activities, mainly antioxidant, anti‐inflammatory, and antimicrobial effects. Isoquercitrin is known for its reported antimicrobial and anticancer activities in previous studies (Mirza et al. 2023), while kaempferol has been linked to antioxidant and anti‐inflammatory effects (Chen et al. 2023). Kaempferol‐3‐glucoside has demonstrated protection against oxidative stress in experimental models (Meng et al. 2019), and fisetin is reported to show antioxidant and neuroprotective potential in preclinical studies (Ravula et al. 2024). Hydroxybenzaldehyde exhibits anti‐inflammatory and anti‐angiogenic effects according to previous reports (Lim et al. 2008), whereas trans‐ferulic acid plays a role in antioxidant defense and inflammation reduction (Zhai et al. 2023). Similarly, p‐coumaric acid is associated with antimicrobial and antioxidant activities (Chen et al. 2024), and caffeic acid shows strong antioxidant as well as anti‐inflammatory potential (Espíndola et al. 2019). Salicylic acid is recognized for its antimicrobial properties and its involvement in plant defense responses (Rosheen et al. 2018). Vanillic acid has been reported also to show antioxidant activities in previous in vitro studies (Ingole et al. 2021). Collectively, these phenolic compounds may contribute to the observed antioxidant and antibacterial activities of the extracts in the present study. The relatively higher total phenolic content of the MeOH extract (26.081 mg/g) compared to ACT (8.809 mg/g) and HEX (0.110 mg/g) further supports its superior antioxidant performance, as many of these compounds may act synergistically in free radical scavenging mechanisms. Moreover, both catechin and hesperidin were detected only in ACT extract. They mentioned in literature for their therapeutics potential. Tea plants are a rich source of catechins, which is widely recognized for having potent antioxidant qualities and ability to protect skin from ultraviolet light. In addition, catechins have antibacterial, antiallergenic, and anti‐inflammatory properties in addition to possible antiviral and anticancer properties (Kumar et al. 2022). Also, the flavonoid hesperidin, which is present in citrus fruits, has antimicrobial, anticancer, anti‐inflammatory, and antioxidant characteristics (Donia et al. 2023). The exclusive presence of these compounds in the ACT extract may explain its stronger antibacterial activity compared to the other extracts, as both catechin and hesperidin are known to disrupt microbial cell membranes and inhibit bacterial growth. Notably, hesperidin was detected at a high concentration (5.447 mg/g), suggesting that it is not only present but may play a significant role in the antibacterial activity observed in the ACT fraction. Furthermore, protocatechuic acid were present exclusively in HEX extract, while polydatin and resveratrol were detected only in MeOH extract. Protocatechuic acid possesses a wide range of pharmacological effects, including anti‐aging, neuroprotective, antibacterial, antiviral, anticancer, anti‐inflammatory, analgesic, antioxidant, and α‐amylase inhibition properties (Aleixandre et al. 2022; Kumar et al. 2022). Given that it is the most abundant identified compound in the HEX extract (0.09 mg.g), it may play a dominant role in the bioactivity of this extract, particularly in its enzymatic inhibition capacity. Both polydatin and resveratrol have gained attention due to their health benefits and biological activities against inflammation and oxidative stress (Karami et al. 2022; Kumar et al. 2022), further reinforcing the strong antioxidant performance of the MeOH extract. Rutin was identified in both ACT and MeOH extracts, it is a plants derived compound shows diverse pharmacological effects, antibacterial, neuroprotective, nephroprotective, hepatoprotective, cardiovascular, and antidiabetic properties (Madkour et al. 2024). Notably, the very high concentration of rutin in the MeOH extract (14.856 mg/g) is expected to play a major role in its superior antioxidant activity, while its moderate presence in ACT (1.849 mg/g) may contribute to the antibacterial and antioxidant effects observed in that fraction. The sequential extraction procedure may explain the observed differences between acetone and methanol extracts, as they are not fully independent. The higher concentration of phenolic compounds in the methanolic extract may be attributed to the greater polarity of methanol, which enhances the extraction of polar secondary metabolites such as phenolic acids and flavonoid glycosides. In addition, incomplete extraction during the acetone step may allow residual compounds to be subsequently recovered by methanol. Therefore, the differences between extracts are likely related to solvent polarity and extraction efficiency.

The Algerian *D. carota* subsp. *maximus* can be considered as a source of high amounts of rutin and chlorogenic acid. In comparison with a previous study, the subspecies *D. carota* subsp. *maximus* presents some resemblance to the wild carrot *D. carota* L. studied by Pavlyuk et al. (2015), which contains seven compounds identified in the current study, namely p‐coumaric acid, caffeic acid, rutin, catechin, trans‐ferulic acid, hydroxybenzaldehyde, and chlorogenic acid. However, when compared with the LC‐ESI‐MS/MS profile of the aqueous extract of *D. carota* subsp. *maximus* reported by Hadji et al. (2024), both similarities and differences were observed. Several compounds were commonly detected in both studies, including chlorogenic acid, caffeic acid, p‐coumaric acid, trans‐ferulic acid, vanillic acid, salicylic acid, hydroxybenzaldehyde, vanillin, quercetin, kaempferol, isoquercitrin, hesperidin, kaempferol‐3‐glucoside, and fisetin. In contrast, the present study additionally identified protocatechuic acid, rutin, polydatin, and resveratrol, which were not detected in the aqueous extract reported by Hadji et al. (2024), whereas gallic acid, syringic acid, sinapic acid, and scutellarin were reported only in that study. Therefore, the present study provides a more comprehensive comparative phytochemical characterization by evaluating multiple solvent extracts, which allows a broader recovery of phenolic constituents and reveals additional bioactive compounds not previously reported under aqueous extraction conditions. This multi‐solvent approach better reflects the chemical diversity of *D. carota* subsp. *maximus* and strengthens the phytochemical understanding of this underexplored plant.

The characterization of phytochemicals from underexplored wild plants may support the identification of new natural sources of bioactive compounds.

### 3.3 In Vitro Antioxidant Activity

In the present study, the antioxidant characteristics of *D. carota* subsp. *maximus* extracts were discovered through various in vitro assays which may provide a significant measure of the plant oxidation inhibition properties. DPPH and ABTS assays were used to evaluate neutralizing and scavenging free radicals' activity. In contrast, CUPRAC, phenanthroline reducing power, potassium ferricyanide reducing power and silver nanoparticles assays evaluate metal ions reducing capacity. Table 3 presents the antioxidant activity results, compared with the reference antioxidants (QCT, BHA, TCP, TX, and AsA), and expressed as IC_50_ and *A*_0.5_ values. The DPPH assay showed that all extracts exhibited antioxidant activity, with the MeOH extract being the most active among the tested samples (IC_50_ = 32.67 ± 0.74 μg/mL), followed by ACT (41.93 ± 0.04 μg/mL) and HEX (128.52 ± 0.33 μg/mL). However, all extracts were significantly less active than the standards BHA (9.69 ± 0.18 μg/mL), TCP (6.75 ± 0.06 μg/mL), and QCT (3.63 ± 0.18 μg/mL). In the ABTS assay, MeOH again showed the strongest activity among extracts (IC_50_ = 13.76 ± 0.06 μg/mL), followed by ACT (17.69 ± 0.34 μg/mL), while HEX displayed weak activity (86.56 ± 0.40 μg/mL). In comparison, both BHA (8.01 ± 0.11 μg/mL) and QCT (< 1.56 μg/mL) were more potent than all extracts. For the CUPRAC assay, the reducing capacity was highest in QCT, followed by TCP, while MeOH and ACT showed similar activity, and HEX exhibited the weakest activity. The *A*_0.5_ values were 2.12 ± 0.04, 17.00 ± 0.37, 47.79 ± 0.12, 48.14 ± 0.26, and 172.13 ± 0.59 μg/mL, respectively, indicating much lower activity in all extracts compared to the standards. Similarly, in the phenanthroline assay, standards (BHA, QCT, and TCP) showed strong reducing power (*A*_0.5_ = 1.84 ± 0.12, 5.40 ± 0.20, and 7.69 ± 1.39 μg/mL, respectively), whereas MeOH and ACT exhibited considerably lower activity (163.67 ± 0.04 and 131.65 ± 1.38 μg/mL, respectively), and HEX exceeded 200 μg/mL, indicating very weak activity. In the potassium ferricyanide assay, MeOH showed the strongest reducing power among extracts (*A*_0.5_ = 36.34 ± 0.83 μg/mL), followed by ACT (68.47 ± 0.87 μg/mL) and HEX (183.33 ± 0.72 μg/mL), although all remained far less active than QCT (1.01 ± 0.24 μg/mL), BHA (1.68 ± 0.37 μg/mL), and TCP (3.46 ± 0.06 μg/mL). Finally, in the silver nitrate (SNP) reducing assay, MeOH exhibited the highest activity among extracts (*A*_0.5_ = 92.92 ± 0.73 μg/mL), followed by ACT (162.75 ± 0.76 μg/mL), while HEX required more than 200 μg/mL to reach 50% reduction. All extracts were markedly less active than ascorbic acid (7.14 ± 0.05 μg/mL) and Trolox (34.17 ± 1.23 μg/mL). The antioxidant activity observed in the present study is in agreement with the moderate activity previously reported for the aqueous extract of *D. carota* subsp. *maximus* by Hadji et al. (2024), although the present organic extracts, particularly methanol, showed higher activity across most assays. The aqueous extract was generally weaker than the organic extracts, highlighting the importance of solvent selection for phenolic compound extraction.

**TABLE 3:** In vitro antioxidant activity of *Daucus carota* subsp. *maximus* organic extracts.

	DPPH	ABTS	CUPRAC	Phenanthroline RP	Potassium ferricyanide RP	SNP
	(IC_50_ μg/mL)			(A_0.5_ μg/mL)		
HEX	128.52 ± 0.33^(f)^	86.56 ± 0.41^(d)^	172.13 ± 0.59^(d)^	> 200	183.33 ± 0.72^(e)^	> 200
ACT	41.93 ± 0.04^(e)^	17.69 ± 0.34^(c)^	48.14 ± 0.26^(c)^	163.67 ± 0.04^(d)^	68.47 ± 0.87^(d)^	162.75 ± 0.76^(d)^
MeOH	32.67 ± 0.74^(d)^	13.76 ± 0.06^(b)^	47.79 ± 0.12^(c)^	131.65 ± 1.38^(c)^	36.34 ± 0.83^(c)^	92.92 ± 0.73^(c)^
QCT	3.63 ± 0.18^(a)^	< 1.5625	2.12 ± 0.04^(a)^	5.40 ± 0.20^(b)^	1.01 ± 0.24^(a)^	NT
BHA	9.69 ± 0.18^(b)^	8.01 ± 0.11^(a)^	NT	1.84 ± 0.12^(a)^	1.68 ± 0.37^(a)^	NT
TCP	6.75 ± 0.06^(c)^	NT	17.00 ± 0.37^(b)^	7.69 ± 1.39^(b)^	3.46 ± 0.06^(b)^	NT
Tx	NT	NT	NT	NT	NT	34.17 ± 1.23^(b)^
AsA	NT	NT	NT	NT	NT	7.14 ± 0.05^(a)^

*Note:* Groups marked with different letters represent values that differ significantly from each other.

Abbreviation: NT: not tested.

In fact, the assessment of *D. carota* subsp. *maximus* antioxidant potential illustrates that the MeOH extract manifested the highest activity with the lowest IC_50_ and *A*_0.5_ values in all assays, followed by the ACT extract, while the hexane extract exhibited the lowest antioxidant activity. Referring to Table 1, the data suggest a relationship between phenolic content and antioxidant IC_50_ and *A*_0.5_ values, indicating that higher phenolic content is associated with stronger antioxidant activity. The results are further supported by the differences in total phenolic quantification by LC‐ESI‐MS/MS, where the MeOH extract exhibited the highest value, followed by ACT extract, while HEX showed the lowest content (Table 2), which is consistent with their respective antioxidant activities. Moreover, the MeOH extract exhibited the highest TFC value, followed by ACT and HEX (Table 1), which is consistent with its superior antioxidant activity. In contrast, although ACT showed relatively high TPC, its lower TFC compared to MeOH may explain its weaker antioxidant performance, suggesting that flavonoid content may play a more important role in antioxidant properties. The extracts' behavior in scavenging free radicals and reducing metal ions may be related to differences in phenolic composition and concentration among all extracts. This variation in phenolic composition and concentrations is mainly related to solvent polarity, which strongly influences the extraction efficiency of phenolic compounds. Polar solvents such as methanol are more effective at extracting phenolic acids, flavonoids, and other antioxidant compounds, whereas less polar solvents like acetone extract moderately polar compounds, and nonpolar solvents such as hexane extract lipophilic constituents that generally exhibit weaker antioxidant activity than phenolic acids and flavonoids (Hadji et al. 2025). As previously mentioned in Table 2, our study has identified some phenolic groups in *D. carota* subsp. *maximus* organic extracts, which vary in their chemical structure and are reported in the literature for their antioxidant properties, such as phenolic acids (Carocho and Ferreira 2013), phenolic aldehydes (Di Majo et al. 2011), stilbenoids (Li et al. 2016), and flavonoids (Naróg and Sobkowiak 2023). Rutin and chlorogenic acid were detected in the MeOH extract with the highest concentrations among all identified compounds in the different extracts. This high content could contribute to the strong antioxidant activity of MeOH, as they are well known for their antioxidant characteristics (Soczynska‐Kordala et al. 2001; Yao et al. 2019; Prakash et al. 2020). Also, both of these compounds are present in the ACT extract in appreciable amounts, which may contribute to its antioxidant activity. Consequently, differences in antioxidant capacity among the extracts are likely related to the type of compounds extracted, their concentrations, and their chemical structures. Numerous researches have emphasized the strong correlation between the chemical structure of phenolic molecules including hydroxyl groups and their position with antioxidant activity (Rice‐Evans et al. 1996; Tung et al. 2009; Yordi et al. 2012; Parcheta et al. 2021). Hydroxyl groups have a wide range of functions, including superoxide radical scavengers, hydrogen donors, and singlet oxygen quenchers (Carocho and Ferreira 2013). More hydroxyl groups enhance the antioxidant potential of phenolic acid compounds (Tung et al. 2009). Specifically, as reported by Parcheta et al. (2021), the most potent antioxidants are derivatives of phenolic acids featuring three hydroxyl groups on the aromatic ring, like gallic acid. Additionally, phenolic acids with two hydroxyl groups positioned on the ortho‐ring, such as protocatechuic acid, exhibit significant antioxidant properties. In contrast, compounds with only one hydroxyl group on an aromatic ring, like 4‐hydroxyphenylacetic acid, demonstrate the lowest antioxidant activity against free radicals.

Chlorogenic acid, a plant secondary metabolite, has potential antioxidant results due to the existence of one carboxyl group and five active hydroxyl groups, which interact with superoxide anions and free hydroxyl radicals (Lafay et al. 2006). In addition to phenolic acids, the strong ability of flavonoids in stabilizing the radical species of compounds is associated with the catechol group on the B‐ring, which easily donates hydrogen or electrons to neutralize this radical species (Procházková et al. 2011). The hydroxyl group at position C3 and C4 of the B‐ring, which forms a catechol moiety, along with a double bond between the second and third carbon atoms and 4‐oxo moieties on the C‐ring, and the presence of a free hydroxyl group at the C3 position in the oxygenated heterocyclic C‐ring, consequently increases the antioxidant capacity of flavonoids compared to isoforms that do not possess these features (Rice‐Evans et al. 1996; Heim et al. 2002). Rutin is a flavone that has antioxidant properties and which can be found in plants (Ganeshpurkar and Saluja 2017). Its potent antioxidant action is due to its distinctive chemical structure, including the presence of a double bond between the second and third carbon atoms linked with 4‐oxo moieties in the C‐ring, the presence of a catechol group on the B‐ring, and a free hyroxhyle group in C3, which is able to bind transition metal ions (Pivec et al. 2019).

Indeed, there is a notable gap in research studies focusing on the variation in extraction solvent polarities to determine the antioxidant properties of *Daucus* species. To our knowledge, this is the first study that highlights free radical scavenging and metal ion reducing properties of *D. carota* subsp. *maximus* using extracts with different polarities. However, one study indicated that 70% ethanol and aqueous *D. carota* L. leaves extracts had a weak activity in scavenging free radicals compared to our results (Kim et al. 2023). Furthermore, ethyl acetate, aqueous, and aqueous/ethyl acetate extracts from *D. carota* Linn seed showed weak DPPH scavenging activity compared to our results (Tijjani et al. 2022). Additionally, another study reported the low reducing capacity of 70% ethanol extract isolated from *D. carota* L. var. *sativa* carrots compared to our findings (Jung et al. 2024).

### 3.4 Antibacterial Potential of *D. carota* Subsp. Maximus

Based on the inhibition zone diameters, the extracts of *D. carota* subsp. *maximus* demonstrated varying levels of antibacterial activity; however, the overall activity can be considered moderate when compared to the antibiotics. ACT extract was effective against all bacterial strains except the negative gram bacteria *E. coli* . The highest activity appeared against *S. aureus* and *M. luteus* , showing an inhibition zone diameter of 14.16 ± 0.28 mm. The extract exhibited an inhibition zone diameter of 14 ± 0.00 mm against *B. subtilis* and 12 ± 0.00 mm against *E. cloacae* and *P. aeruginosa* .

HEX extract showed the highest zone inhibition against *S. aureus* and *P. aeruginosa* (12.16 ± 0.1 and 12 ± 0.00 mm, in order). For *B. subtilis* , HEX extract shows a small inhibition zone (10.20 ± 0.35 mm). Additionally, no activity was observed against positive gram bacteria *M. luteus* , negative gram bacteria *E. coli* , and *E. cloacae* .

**FIGURE 4:**
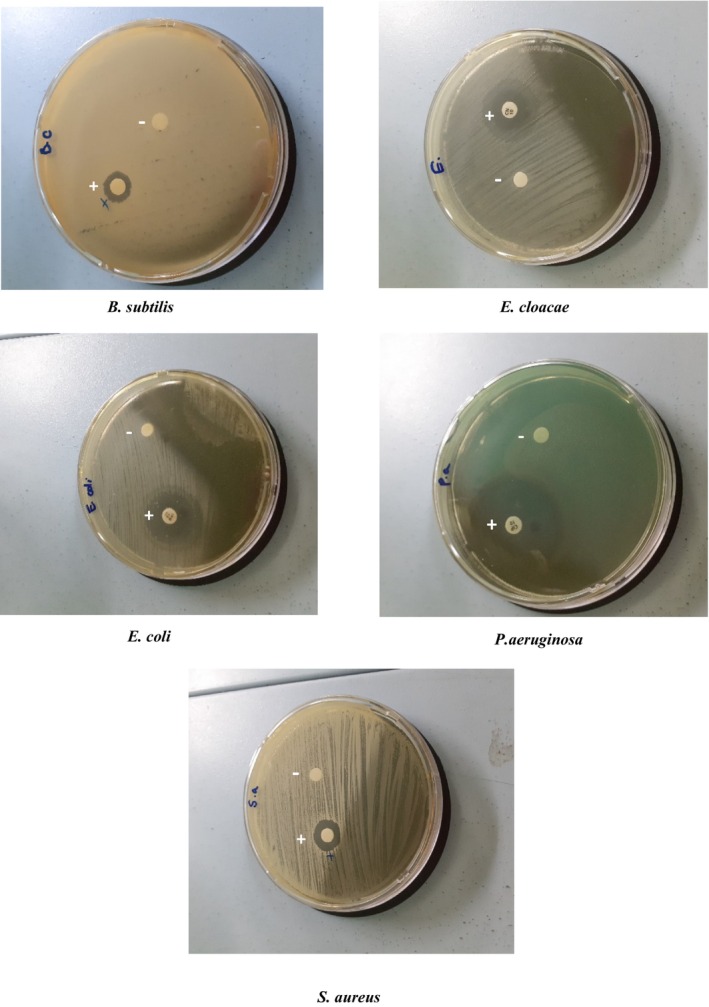
Representative photographs of the positive and negative controls against the tested bacterial strains. (+) Positive control (antibiotic), showing a clear inhibition zone; (−) Negative control (5% DMSO), showing no inhibition zone. The photographs represent the original experimental records. The original photograph for *Micrococcus luteus* is unavailable; however, the 5% DMSO negative control consistently showed no antibacterial activity.

MeOH extract activity was only recorded against two positive bacterial strains *B. subtilis* *and S. aureus*, in which the inhibition zone diameter was 14 ± 0.00 mm, and one negative gram bacteria *P. aeruginosa* with an inhibition zone diameter of 10 ± 0.00 mm against. In contrast, 5% DMSO demonstrated no activity against all of the tested bacterial strains. Also, all antibiotics had a strong inhibition activity against the specific strains, in which the inhibition zone diameter ranges from 14 to 22 mm as demonstrated in Table 4 (Figure 4).

**TABLE 4:** Antibacterial activity of *Daucus carota* subsp. *maximus* organic extracts using agar diffusion method.

Inhibition zone diameter (mm)								
Bacterial strains	HEX	ACT	MeOH	VN	CIP	CN	IMP	DMSO
	200 mg/mL			10 μg/mL				5%
*Bacillus subtilis*	10.20 ± 0.35^(a)^	14 ± 00^(b)^	14 ± 00^(b)^	14 ± 00^(c)^	NT	NT	NT	NA
*Staphylococcus aureus*	12.16 ± 0.1^(b)^	14.16 ± 0.28^(b)^	14 ± 00^(b)^	NT	NT	16 ± 00^(c)^	NT	NA
*Enterobacter cloacae*	NA	12 ± 00^(b)^	NA	NT	18 ± 00^(a)^	NT	NT	NT
*Escherichia coli*	NA	NA	NA	NT	NT	NT	20 ± 00	NA
*Pseudomonas aeruginosa*	12 ± 00^(b)^	12 ± 00^(b)^	10 ± 00^(a)^	NT	22 ± 00^(c)^	NT	NT	NA
*Micrococcus luteus*	NA	14.16 ± 0.28^(a)^	NA	16 ± 00^(b)^	NT	NT	NT	NA

*Note:* Groups marked with different letters represent values that differ significantly from each other.

Abbreviations: NA, no activity; NT, not tested.

The MIC and MBC of *D. carota* subsp. maximus extracts evaluated for all extracts that have been shown antibacterial susceptibility (Table 5). The analysis of results shows that, with *P. aeruginosa* and *M. luteus* , only ACT extract had minimum inhibitory concentrations of less than 1 mg/mL (MIC: 0.78 ± 00 mg/mL). For *P. aeruginosa* and *M. luteus* , MBC values were identified as 1.56 and 3.125 mg/mL, respectively.

**TABLE 5:** Minimum inhibitory concentrations and minimum bactericidal concentrations of *Daucus carota* subsp. *maximus* organic extracts.

Bacterial strains	Samples	CMI	MBC
		(mg/mL)	
*Bacillus subtilis*	HEX	50 ± 0.00	100 ± 0.00
	ACT	6.25 ± 0.00	12.5 ± 0.00
	MeOH	12.5 ± 0.00	25 ± 0.00
	G	< 0.019	—
*Staphylococcus aureus*	HEX	25 ± 0.00	50 ± 0.00
	ACT	6.25 ± 0.00	12.5 ± 0.00
	MeOH	6.25 ± 0.00	12.5 ± 0.00
	G	< 0.019	—
*Enterobacter cloacae*	HEX	NT	NT
	ACT	25 ± 0.00	50 ± 0.00
	MeOH	NT	NT
	G	< 0.019	—
*Pseudomonas aeruginosa*	HEX	3.125 ± 0.00	6.25 ± 0.00
	ACT	0.78 ± 0.00	1.56 ± 0.00
	MeOH	3.125 ± 0.00	6.25 ± 0.00
	G	< 0.019	—
*Micrococcus luteus*	HEX	NT	NT
	ACT	0.78 ± 0.00	3.125 ± 0.00
	MeOH	NT	NT
	G	< 0.019	—

Abbreviations: G, gentamicin; NT, not tested.

The *S. aureus* strain was suppressed by the MeOH and ACT extracts at concentrations of 6.25 mg/mL and eliminated at 12.5 mg/mL. Regarding *B. subtilis* strain, the minimum inhibitory concentrations were 50, 6.25, and 12.5 mg/mL for HEX, ACT and MeOH extracts, in order. Furthermore, the minimum bactericidal concentrations were 100, 12.5 and 25 mg/mL for the same extracts, in order.

ACT inhibited *E. cloacae* at 25 mg/mL. Additionally, it exterminated it at 50 mg/mL. The minimal inhibitory and bactericidal concentrations for *P. aeruginosa* were found in HEX and MeOH extracts to be 3.125 and 6.25 mg/mL, respectively.

All extracts showed lower inhibitory concentrations compared to gentamicin, which was used as the standard antibiotic with MICs lower than 0.019 mg/mL. Altogether, the MIC and MBC results supported the agar diffusion results, identifying the acetone extract as the most active extract against both Gram‐positive and Gram‐negative bacteria, except *E. coli* . Nevertheless, the relationship between inhibition zone diameters, MIC and MBC values was not always direct, as some extracts exhibiting comparable inhibition zones showed differences in their inhibitory and bactericidal concentrations. Therefore, MIC and MBC values provide a more quantitative assessment of antibacterial activity than inhibition zone diameters alone. This outcome suggests that acetone extraction may have yielded a higher concentration and broader spectrum of antibacterial phytochemicals, resulting in stronger activity against the tested bacterial strains compared to *n*‐hexane and methanol extracts. Several studies have reported the effectiveness of acetone in extracting antimicrobial compounds from plant materials compared with other solvents such as methanol, chloroform, and hexane (Eloff 1998). Acetone, as a solvent of intermediate polarity, is capable of extracting both polar and moderately nonpolar compounds, which may act individually or synergistically to enhance antibacterial activity. In contrast, methanol primarily extracts highly polar constituents, whereas hexane mainly recovers non‐polar lipophilic compounds, which may contribute differently to antibacterial effects (Kotzé and Eloff 2002; Eloff 1998). This broader extraction profile of acetone may explain the stronger antibacterial activity observed in the acetone extract. It can be hypothesized that hesperidin, identified as a major compound and previously reported to possess antibacterial properties (Choi et al. 2022), contributes to this activity, as it is present only in the ACT extract; however, further phytochemical characterization and bioactivity‐guided fractionation are necessary to confirm its specific contribution. Moreover, the observed pattern of bacterial susceptibility is influenced by the structural differences between Gram‐positive and Gram‐negative bacteria. Gram‐negative bacteria possess an outer membrane with a lipopolysaccharide layer that restricts the penetration of many antibacterial compounds, including plant‐derived molecules, particularly polar and highly charged compounds such as those extracted by methanol, whereas neutral, less polar and lipophilic compounds extracted by acetone or *n*‐hexane can penetrate more easily (Saxena et al. 2023). In contrast, Gram‐positive bacteria, which lack this outer barrier, are more susceptible to antibacterial compounds from all extracts (Gregory and Langland 2025).

The antibacterial efficacy of species from the genus *Daucus* has rarely been evaluated using solvents with different polarities. To date, the antibacterial potential of *D. carota* subsp. *maximus* has been studied only for essential oils. Therefore, this is the first study evaluating the antibacterial properties of organic extracts from this subspecies. A previous study on *D. carota* L. leaves indicated that the ethyl acetate fraction exhibited the highest activity against *S. aureus* , comparable to gentamicin, while water, *n*‐hexane, and 96% ethanol fractions were less effective (Akune et al. 2024). These findings align with our results, highlighting that solvents with moderate polarity are more efficient in isolating antibacterial compounds than strongly polar or nonpolar solvents.

### 3.5 In Vitro Alpha Amylase Inhibition Activity

In the current study, alpha amylase inhibition activity of *D. carota* subsp. *maximus* extracts was assessed through determining the concentration needed to inhibit 50% of this enzyme (IC_50_), compared to acarbose. According to the results displayed in Table 6, HEX extract exhibits the highest inhibitory percentage among all extracts, with a value of 52.15% ± 0.23% at a concentration of 400 μg/mL, similar to acarbose, which has an inhibition rate of 53.05% ± 1.59% at the same concentration, with no significant difference between the two.

**TABLE 6:** Alpha amylase inhibition activity of *Daucus carota* subsp. *maximus* extracts.

Concentration (μg/mL)	α‐amylase Inhibition %			
	HEX	ACT	MeOH	Acarbose
400	52.15 ± 0.23^(l)^	28.45 ± 0.18^(h)^	6.63 ± 0.18^(c)^	53.05 ± 1.59^(l)^
200	25.09 ± 0.27^(g)^	23.34 ± 0.19^(f)^	3.12 ± 0.12^(b)^	37.21 ± 3.54^(j)^
100	13.33 ± 0.09^(e)^	12.43 ± 0.62^(d)^	0.93 ± 0.13^(a)^	31.81 ± 2.89^(i)^
IC_50_ (μg/mL)	381.07 ± 0.52^(a)^	> 400	> 400	365.93 ± 1.70^(b)^

*Note:* Different superscript letters indicate significant differences among all samples and concentrations for inhibition data, and among IC_50_ values of the tested samples.

The IC_50_ value of the HEX extract is 381.07 ± 0.52 μg/mL, which is close to acarbose, with an IC_50_ of 365.93 ± 1.70 μg/mL.

The remaining extracts exhibit insufficient inhibitory effects, with IC_50_ values exceeding 400 μg/mL. The ACT extract demonstrated an inhibition percentage of 28.45% ± 0.18%, while the MeOH showed weak activity, with maximum inhibition rates of 6.63% ± 0.18% at 400 μg/mL.

However, among the phenolic compounds identified in the HEX extract, protocatechuic acid was unique, as vanillin and chlorogenic acid were also present in the other extracts. Protocatechuic acid has been reported to inhibit α‐amylase activity (Aleixandre et al. 2022), which may account for the relatively higher inhibitory effect observed in the HEX extract.

The differences in activity among the extracts can be largely attributed to the solvent‐dependent extraction of phytochemicals. *n*‐Hexane, being a non‐polar solvent, selectively extracts lipophilic compounds such as lipids, terpenoids, and aromatic hydrocarbons, which are often associated with enzymatic inhibition (Cravotto et al. 2022). Although the specific lipophilic compounds in *D. carota* subsp. *maximus* were not directly identified in the current study, the high α‐amylase inhibitory activity of the HEX extract suggests that such non‐polar bioactive compounds were likely extracted by *n*‐hexane solvent. Previous studies have reported that *n*‐hexane extracts from various plants exhibit the highest α‐amylase inhibitory activity due to the presence of lipophilic compounds, whereas polar solvents preferentially extract hydrophilic compounds, such as flavonoids, glycosides, and certain phenolic acids, which are more commonly associated with antioxidant and antimicrobial activities (Vadivelan et al. 2018; Mogole et al. 2020).

Given the reviewed literature, this is the first study on this subspecies to evaluate α‐amylase inhibition activity. A previous study reported that polar and weakly polar extracts (aqueous and ethyl acetate) from *D. carota* seeds are effective inhibitors of α‐amylase (Tijjani and Imam 2021), which differs from our findings.

### 3.6 Bioavailability and Pharmacokinetics

**FIGURE 5:**
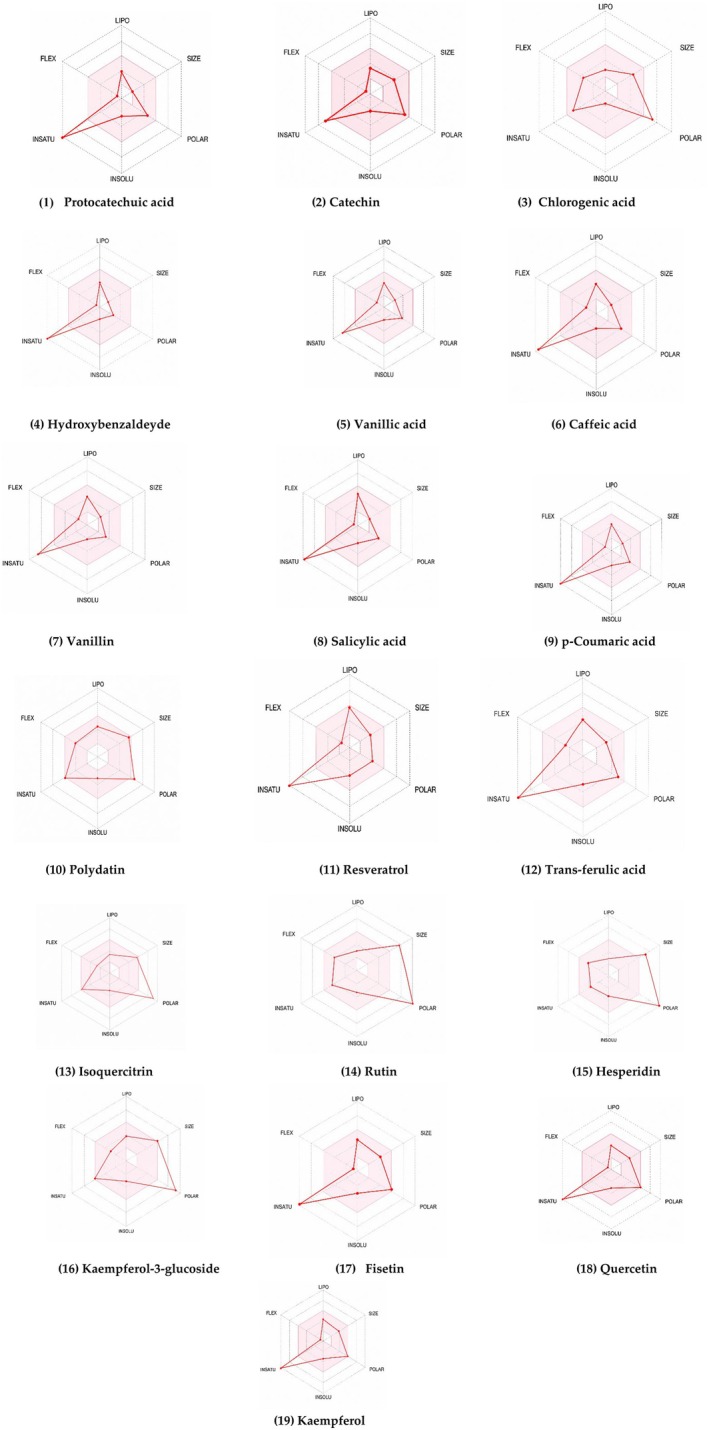
Bioavailability hexagons for the main compounds identified in *Daucus carota* subsp. *maximus* organic extracts through LC‐ESI‐MS/MS analysis (FLEX: Flexibility, SIZE: Molecular size, POLA: Polarity, INSA: Unsaturation, INSO: Insolubility, LIPO: Lipophilicity). Listed compounds from 1 to 19.

The bioavailability and pharmacokinetic analyses are useful for predicting drug likeness and guiding further experimental studies, as these properties determine how effectively compounds are absorbed, distributed, metabolized, and excreted in the body (Price and Patel 2023). In this study, the bioavailability and pharmacokinetic properties of the compounds identified via LC‐ESI‐MS/MS in *D. carota* subsp. *maximus* organic extracts were analyzed and presented in Figure 5, Tables 7 and 8.

**TABLE 7:** Physicochemical properties and lipophilicity of the identified compounds in *Daucus carota* subsp. *maximus* organic extracts.

Molecule	1	2	3	4	5	6	7	8	9	10	11	12	13	14	15	16	17	18	19
Molecular weight	154.12	290.27	354.31	122.12	168.15	180.16	152.15	138.12	164.16	390.38	228.24	194.18	464.38	610.52	610.56	448.38	286.24	302.24	286.24
No. Heavy atoms	11	21	25	9	12	13	11	10	12	28	17	14	33	43	43	32	21	22	21
No. Aromatic heavy atoms	6	12	6	6	6	6	6	6	6	12	12	6	16	16	12	16	16	16	16
Fraction Csp3	0	0.2	0.38	0	0.12	0	0.12	0	0	0.3	0	0.1	0.29	0.44	0.54	0.29	0	0	0
No. Rotatable bonds	1	1	5	1	2	2	2	1	2	5	2	3	4	6	7	4	1	1	1
No. H‐bond acceptors	4	6	9	2	4	4	3	3	3	8	3	4	12	16	15	11	6	7	6
No. H‐bond donors	3	5	6	1	2	3	1	2	2	6	3	2	8	10	8	7	4	5	4
Molecular refractivity	37.45	74.33	83.5	33.85	41.92	47.16	40.34	35.42	45.13	100	67.88	51.63	110.16	141.38	141.41	108.13	76.01	78.03	76.01
TPSA (Å^2^)	77.76	110.38	164.75	37.3	66.76	77.76	46.53	57.53	57.53	139.84	60.69	66.76	210.51	269.43	234.29	190.28	111.13	131.36	111.13
Consensus Log *P*_o/w_	0.65	0.85	−0.38	1.17	1.08	0.93	1.2	1.24	1.26	0.62	2.48	1.36	−0.25	−1.29	−0.72	−0.25	1.55	1.23	1.58

**TABLE 8:** Druglikeness, bioavailability, and pharmacokinetic profiles of the identified compounds in *Daucus* *carota* subsp. *maximus* organic extracts.

Molecule	1	2	3	4	5	6	7	8	9	10	11	12	13	14	15	16	17	18	19
GI absorption	High	High	Low	High	High	High	High	High	High	High	High	High	Low	Low	Low	Low	High	High	High
BBB permeant	No	No	No	Yes	No	No	Yes	Yes	Yes	No	Yes	Yes	No	No	No	No	No	No	No
Pgp substrate	No	Yes	No	No	No	No	No	No	No	Yes	No	No	No	Yes	Yes	No	No	No	No
CYP1A2 inhibitor	No	No	No	No	No	No	No	No	No	No	Yes	No	No	No	No	No	Yes	Yes	Yes
CYP2C19 inhibitor	No	No	No	No	No	No	No	No	No	No	No	No	No	No	No	No	No	No	No
CYP2C9 inhibitor	No	No	No	No	No	No	No	No	No	No	Yes	No	No	No	No	No	No	No	No
CYP2D6 inhibitor	No	No	No	No	No	No	No	No	No	No	No	No	No	No	No	No	Yes	Yes	Yes
CYP3A4 inhibitor	Yes	No	No	No	No	No	No	No	No	No	Yes	No	No	No	No	No	Yes	Yes	Yes
log Kp (cm/s)	−6.42	−7.82	−8.76	−6.09	−6.31	−6.58	−6.37	−5.54	−6.26	−7.95	−5.47	−6.41	−8.88	−10.26	−10.12	−8.52	−6.65	−7.05	−6.7
Bioavailability score	0.56	0.55	0.11	0.55	0.85	0.56	0.55	0.85	0.85	0.55	0.55	0.85	0.17	0.17	0.17	0.17	0.55	0.55	0.55
Synthetic accessibility	1.07	3.5	4.16	1	1.42	1.81	1.15	1	1.61	4.82	2.02	1.93	5.32	6.52	6.34	5.29	3.16	3.23	3.14

Our data showed that most of the compounds exhibited favorable bioavailability scores (BAS), with values ranging between 0.11 and 0.85. Compounds such as salicylic acid, p‐coumaric acid, and caffeic acid demonstrated relatively high bioavailability scores, indicating their potential effectiveness when administered orally. This was additionally confirmed by the bioavailability hexagons (Figure 5), which are influenced by the physical and chemical properties of the compounds. On the other hand, compounds like chlorogenic acid and rutin had lower bioavailability scores, suggesting that their therapeutic potential needs a higher dose to attain the minimum effective concentration level (Price and Patel 2023). Additionally, gastrointestinal (GI) absorption was predicted to be high for most of the compounds. Phytochemicals like caffeic acid, vanillin, and salicylic acid were shown to have high GI absorption, suggesting possible oral absorption, although this requires experimental verification. Regarding blood–brain barrier (BBB) permeability, only hydroxybenzaldehyde, vanillin, salicylic acid, p‐coumaric acid, resveratrol, and trans‐ferulic acid demonstrated the ability to cross the BBB. This suggests that these compounds may have the ability to cross the BBB, although further in vivo studies are required to confirm their potential CNS‐related applications. Most of the other compounds, including rutin, isoquercitrin, and kaempferol, did not permeate the BBB, suggesting that their effects are likely limited to peripheral actions rather than CNS‐related therapeutic benefits.

The skin permeability of the identified compounds was moderate to low, with log Kp values ranging between −5.47 cm/s (for resveratrol) and −10.26 cm/s (for rutin). These results suggest that while some compounds might exhibit potential for transdermal delivery, others may face challenges penetrating the skin. For instance, resveratrol and salicylic acid showed better skin permeability compared to more polar compounds like rutin and isoquercitrin, indicating their possible use in topical formulations for skin‐related issues. Moreover, none of the compounds exhibited significant inhibition of cytochrome P450 (CYP) enzymes such as CYP1A2, CYP2C19, and CYP2C9, with the exception of resveratrol, fisetin, kaempferol, and quercetin, which showed inhibitory effects on CYP2D6 and CYP3A4. In addition, protocatechuic acid exhibited an inhibition effect only for CYP3A4. These findings may indicate potential drug interaction risks in silico and require experimental confirmation, as inhibition of CYP3A4 can lead to altered drug metabolism and potential side effects when combined with other clinical drugs (Guttman and Kerem 2022). However, the inhibitory effects of these compounds can vary based on concentration (Lee et al. 2024).

Most of the compounds were not P‐glycoprotein (P‐gp) substrates, meaning they are unlikely to be effluxed out of cells, which supports their bioavailability and cellular retention.

The synthetic accessibility of the compounds varied, with values ranging from 1.0 (for compounds like protocatechuic acid and hydroxybenzaldehyde) to 6.52 (for rutin), indicating that while some compounds are easy to synthesize, others may require more complex chemical synthesis procedures. Despite these variations, most of the compounds fall within an accessible range, making them feasible candidates for further drug development.

**FIGURE 6:**
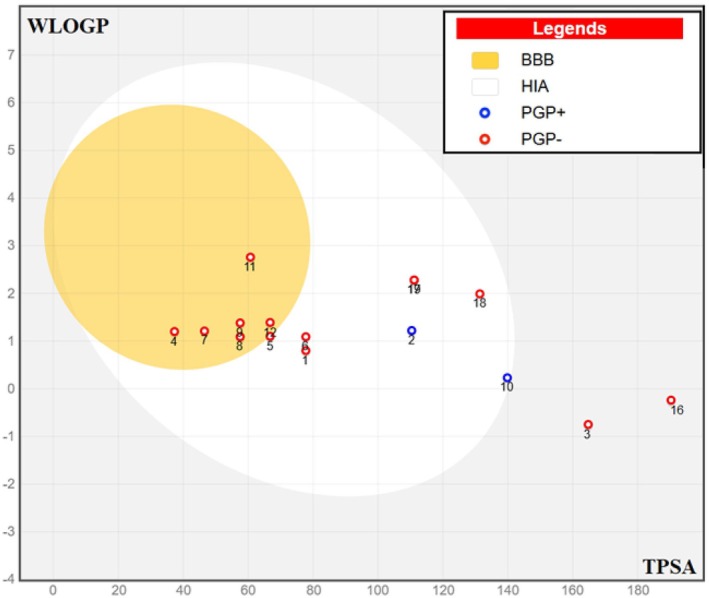
Boiled‐egg model of the compounds identified by LC‐ESI‐MS/MS in *Daucus carota* subsp. *maximus* organic extracts: (1) Protocatechuic acid, (3) Chlorogenic acid, (4) Hydroxybenzaldehyde, (5) Vanillic acid, (6) Caffeic acid, (7) Vanillin, (8) Salicylic acid, (9) p‐Coumaric acid, (11) Resveratrol, (12) trans‐Ferulic acid, (16) Kaempferol‐3‐glucoside, (17) Fisetin, (18) Quercetin, (19) Kaempferol.

The BOILED‐Egg model analysis supported the predicted gastrointestinal absorption, blood–brain barrier permeability, human intestinal absorption, and P‐glycoprotein substrate profiles of the identified compounds, as shown in Figure 6.

In vitro biological activity together with in silico ADME predictions provides preliminary insights into the pharmacological relevance of the identified compounds in *D. carota* subsp. maximus organic extracts.

## 4 Conclusions

This study presents a comprehensive assessment of the phytochemical composition and biological activities of *D. carota* subsp. *maximus* extracts prepared using solvents of different polarities. The results indicate that solvent polarity strongly influences the extraction of bioactive constituents and their associated bioactivities. LC‐ESI‐MS/MS analysis showed that the methanolic extract provided the highest mass recovery and the greatest abundance of major phenolic compounds, which was associated with stronger antioxidant activity. In contrast, the acetone extract contained the highest total phenolic content and showed broader antibacterial activity, while the hexane extract exhibited the most pronounced α‐amylase inhibitory effect. Complementing these in vitro findings, in silico ADME analysis showed that most identified compounds possess favorable gastrointestinal absorption and acceptable bioavailability, indicating potential suitability for oral use. Taken together, these findings highlight the importance of solvent polarity as a key determinant in revealing the full phytochemical and biological potential of plant materials. The observed variability in antioxidant, antibacterial, and α‐amylase inhibitory activities among the different extracts demonstrates that distinct solvent systems selectively enrich different bioactive constituents, each contributing to specific functional properties. In particular, the strong antioxidant activity of the methanolic extract, the broad antibacterial efficacy of the acetonic extract, and the pronounced α‐amylase inhibitory activity of the hexanic extract collectively underscore the complementary nature of the extracted phytochemical profiles. Moreover, the favorable predicted pharmacokinetic properties of most compounds further support their possible applicability for oral bioactive formulations, strengthening the potential of *D. carota* subsp. *maximus* as a promising source of multifunctional natural agents for pharmaceutical, nutraceutical, and food related uses. Although LC‐ESI‐MS/MS profiling enabled the identification of major phenolic constituents in *D. carota* subsp. *maximus*, minor compounds and non‐phenolic metabolites may have remained undetected, suggesting the need for complementary analytical approaches to achieve a more comprehensive metabolomic characterization. The observed biological activities of the crude extracts highlight the contribution of complex phytochemical matrices, yet the potential synergistic or antagonistic interactions among constituents remain insufficiently understood and warrant further investigation through activity guided fractionation and compound isolation to determine whether the effects are driven by individual bioactive compounds or synergistic combinations. In parallel, the biological evaluations, including both in vitro assays and in silico ADME predictions in the current study, were limited to preliminary approaches that cannot fully replicate complex in vivo physiological conditions. While the in silico analyses provided valuable initial insights into the pharmacokinetic behavior of the identified compounds, these predictions require experimental validation to confirm their reliability. Consequently, further in vivo, cellular, and clinical studies are necessary to evaluate toxicity and verify safety, metabolic fate, and therapeutic efficacy, as well as to support the selection of promising candidates for formulation development and optimization of biological performance.

The biological activities observed in *D. carota* subsp. *maximus* highlight its potential as a valuable natural resource for the development of functional foods, nutraceuticals, natural antioxidants, and therapeutic agents targeting oxidative stress, microbial infections, and metabolic disorders. In this context, the strong antioxidant activity of the methanolic and acetonic extracts suggests their suitability for use as plant‐based preservatives in the food industry, where they could help limit lipid oxidation, preserve nutritional quality, and extend the shelf life of fat‐rich food products. Likewise, the moderate but broad‐spectrum antibacterial activity of the acetonic extract supports its possible use as a natural ingredient in functional foods, nutraceutical formulations, and cosmeceutical products aimed at reducing oxidative stress and controlling microbial contamination. However, further studies including food matrix testing, toxicity evaluation, sensory analysis, stability studies, and biofilm assays are required to fully validate these applications. In addition, the α‐amylase inhibitory activity exhibited by the HEX extract indicates the presence of compounds with potential relevance to postprandial glycemic control, pending confirmation through enzyme kinetic, cellular, and in vivo studies. These findings suggest possible applications in the development of functional foods or nutraceuticals targeting postprandial glycemic regulation. The results highlight the importance of this wild subspecies and support further studies aimed at evaluating its therapeutic and industrial applications.

## Author Contributions

**Khadidja Dehimi:** methodology, resources. **Maroua Hadji:** conceptualization, investigation, methodology, writing – original draft. **Tarek H. Taha:** formal analysis, project administration. **Fehmi Boufahja:** investigation. **Hamdi Bendif:** funding acquisition, supervision, writing – review and editing, project administration. **Walid Elfalleh:** formal analysis, visualization. **Stefania Garzoli:** supervision, writing – review and editing. **Ilyas Yildiz:** investigation, methodology, visualization. **Toka Hadji:** formal analysis, software, writing – original draft. **Fethi Farouk Kebaili:** investigation, resources. **Tahar Smaili:** conceptualization. **Larbi Derbak:** investigation, methodology, writing – original draft.

## Funding

This work was supported and funded by the Deanship of Scientific Research at Imam Mohammad Ibn Saud Islamic University (IMSIU) (grant number IMSIU‐DDRSP2601).

## Conflicts of Interest

The authors declare no conflicts of interest.

## Data Availability

Data will be made available on request.
